# Immune response to sympatric and allopatric parasites in a snail-trematode interaction

**DOI:** 10.1186/1742-9994-2-8

**Published:** 2005-05-31

**Authors:** Erik E Osnas, Curtis M Lively

**Affiliations:** 1 Department of Biology, Indiana University, 1001 E. Third Street, Bloomington, IN 47405, USA; 2 Department of Wildlife Ecology, University of Wisconsin, 209 Russell Labs, 1630 Linden Drive, Madison, WI 53706, USA

**Keywords:** coevolution, local adaptation, immune defense, trematode

## Abstract

**Background:**

The outcome of parasite exposure depends on the (1) genetic specificity of the interaction, (2) induction of host defenses, and (3) parasite counter defenses. We studied both the genetic specificity for infection and the specificity for the host-defense response in a snail-trematode interaction (*Potamopyrgus antipodarum-Microphallus *sp.) by conducting a reciprocal cross-infection experiment between two populations of host and parasite.

**Results:**

We found that infection was greater in sympatric host-parasite combinations. We also found that the host-defense response (hemocyte concentration) was induced by parasite exposure, but the response did not increase with increased parasite dose nor did it depend on parasite source, host source, or host-parasite combination.

**Conclusion:**

The results are consistent with a genetically specific host-parasite interaction, but inconsistent with a general arms-race type interaction where allocation to defense is the main determinant of host resistance.

## Background

Studies of host-parasite interactions can be seen as split between two different approaches [1,2]. One approach tends to emphasize the induction and cost of defense against parasites, while the other approach tends to emphasize the genetic basis and specificity required for successful infection. Both avenues have been productive, but they need not be seen as mutually exclusive [1-3]. The induction of an immune defense, for example, might be required to eliminate parasites once detected by the host [4]. On the other hand, the relative effectiveness of host defense is expected to decline as the diversity of parasite genotypes increases, provided these genotypes show a high degree of host specificity [1]. Understanding the relative importance and possible interactions between immune defense and genotypic specificity requires that both aspects are studied simultaneously in the same system. For example, Kurtz et al. [5] showed that the immune response of grasshoppers was reduced in foreign environments, even though body mass, a measure of general condition, was not reduced. They interpreted this to mean that the grasshoppers require less immune defense in the face of foreign, and presumably locally adapted, parasites. In order to more fully address the interaction between genetic specificity and immune defense, one could first test for local adaptation, and then measure the immune response in both sympatric and allopatric host-parasite combinations.

In the present study, we exposed host snails from two lake populations to two different doses of eggs produced by either sympatric or allopatric trematodes in a reciprocal cross-infection experiment. We then compared the two different egg doses to each other and to no-egg controls to determine whether the host immune system could be induced, and, if so, whether it depended on parasite dose and/or source (i.e., sympatric vs. allopatric). We found that the immune system could be induced to increase the number of hemocytes, but that the induction did not depend on dose or the source of parasites. Nonetheless, the prevalence of infection was greater for sympatric combinations of host and parasite. Thus the induction of defense was not as effective at combating the apparently co-evolved parasites as it was in combating the remote sources of parasites.

## Results

**Figure 1:**
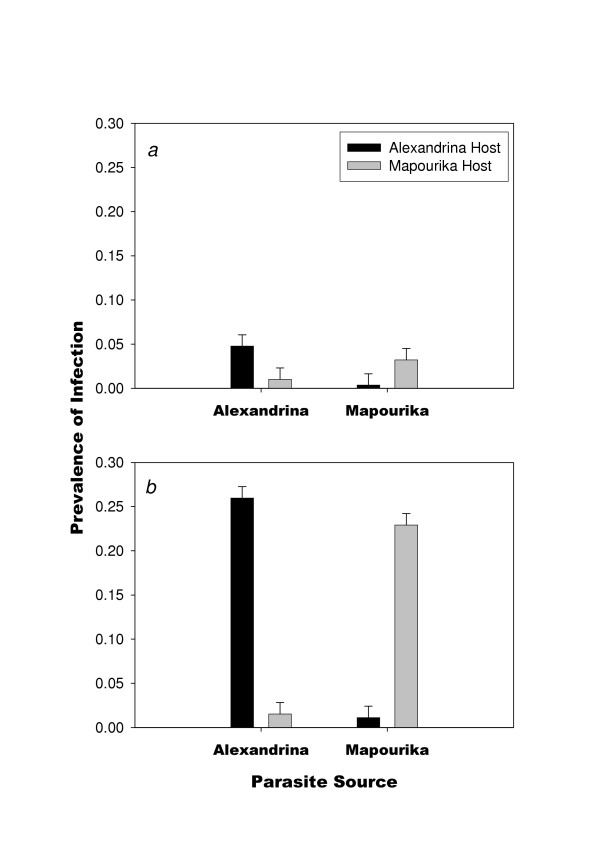
Mean prevalence *Microphallus *infection (1 s.e.) of the snail host *P. antipodarum *in relation to parasite source, host source, and dose of parasite: (*a*) low dose, and (*b*) high dose. Vertical standard error bars were estimated from the mean squared error in the ANOVA in Table 1.

There were no significant host-source or parasite-source main effects on parasite prevalence, but a significant main effect of parasite dose was observed (*F *= 136.08, d.f. = 1, 40, *P *< 0.001; Table 1; Fig. 1). The two-way interaction between host source and parasite source was significant (*F *= 213.66, d.f. = 1, 40, *P *< 0.001), as was the three-way interaction between host source, parasite source, and parasite dose (*F *= 120.09, d.f. = 1, 40, *P *< 0.001; Table 1; Fig. 1). No other interactions were significant. The form of the two-way interaction between host and parasite is of the crossing type, indicating that parasites produced higher prevalence of infection in sympatric hosts than in allopatric hosts (Fig. 1a and 1b). The significant three-way interaction between dose, host, and parasite indicates that the strength of the two-way interaction (local adaptation of the parasite) increased with dose (Fig. 1a vs. 1b).

**Table 1:** Fixed-effect analysis of variance for prevalence of infection with parasite source, host source, and parasite dose as factors. R-squared for the model was 0.92.

Source	s.s.	d.f.	m.s.	*F*	*P*
Parasite source	0.002	1	0.002	2.45	0.125
Host source	0.001	1	0.001	0.96	0.333
Dose	0.133	1	0.133	136.08	<0.001
Parasite * Host	0.210	1	0.210	213.66	<0.001
Parasite * Dose	<0.001	1	<0.001	0.12	0.727
Host * Dose	<0.001	1	<0.001	0.24	0.629
Parasite * Host * Dose	0.118	1	0.118	120.09	<0.001
Error	0.039	40	0.001		
Total	0.782	48			

**Figure 2:**
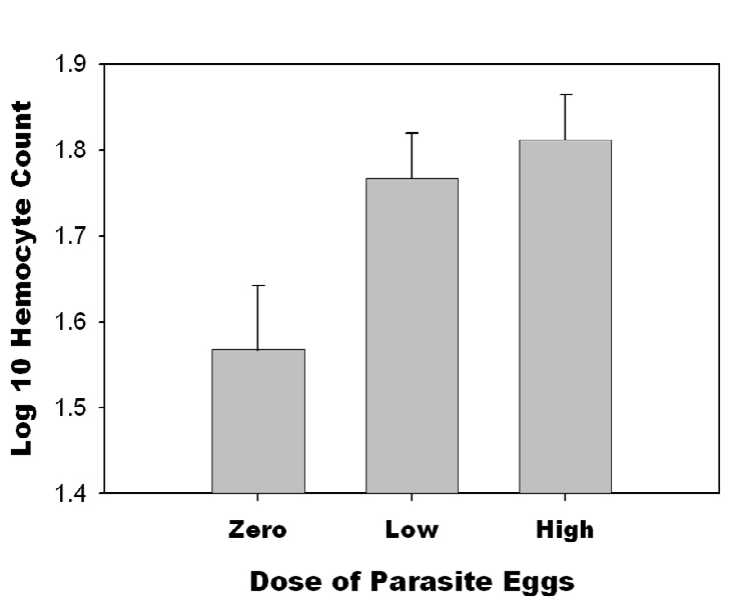
Mean hemocyte count (1 s.e.) in 0.1 μl hemolymph of the snail host *P. antipodarum *in relation to dose of parasite. Vertical standard error bars were estimated from the mean squared error in the ANOVA in Table 2.

**Figure 3:**
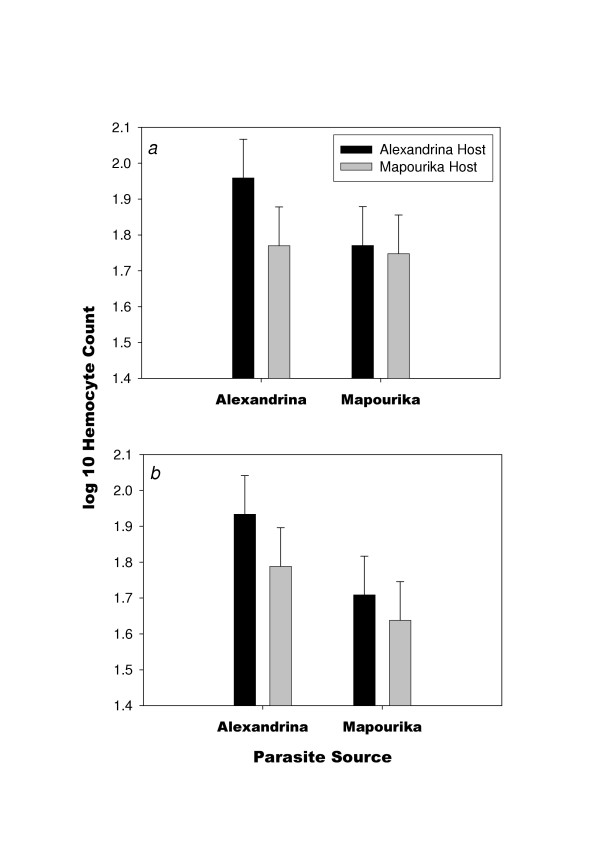
Mean hemocyte count (1 s.e.) in 0.1 μl hemolymph of the snail host *P. antipodarum *in relation to parasite source, host source, and dose of parasite: (*a*) low dose, and (*b*) high dose. Vertical standard error bars were estimated from the mean squared error in the ANOVA in Table 3

There was a significant effect of parasite exposure on hemocyte count (*F *= 3.71, d.f. = 2, 57, *P *= 0.03; Table 2; Fig. 2). Hemocyte count increased with exposure to parasites (*t *= 2.66, *P *= 0.01), but there was not a significant difference between low and high doses of parasites (*t *= 0.66, *P *= 0.55; Table 2; Fig. 2). When the controls (no exposure) were deleted and the data set was analyzed as a three-factor experiment, there was only a marginally significant effect of parasite source on hemocyte count (*F *= 3.69, d.f. = 1, 40, *P *= 0.06); no other effects or interactions were significant (Fig. 3, Table 3). This marginally significant result suggests that parasites from L. Alexandrina tended to induce a slightly higher hemocyte count than did parasites from L. Mapourika (Fig. 3). Observed power estimates for the effects of dose, parasite source, host source, and the interaction between host and parasite were low (0.08, 0.48, 0.27, and 0.12, respectively). Observed power estimates for the other interactions were also low (< 0.08). However, observed effect sizes were also small (<10%) for all effects. When larger effect sizes were used to estimate power (10% and 20%), the power to detect an effect was moderately high (0.55 and 0.88, respectively). Therefore, it is likely that the true effect sizes were less than 10%.

**Table 2:** One-way analysis of variance for average hemocyte count across three parasite dose treatments (zero, low, and high). The first contrast tests for a difference between exposed (low and high dose) and not exposed treatments (zero dose), and the second contrast tests for a difference between low and high dose treatments.

		s.s.	d.f.	m.s.	*F*	*p*
Between Groups		0.496	2	0.248	3.705	0.031
Within Groups		3.819	57	0.067		
Total		4.316	59			
	
	Contrast			*t*	d.f.	*p*
	Control vs. Exposed			2.66	57	0.010
	Low dose vs. High dose			0.600	57	0.551

**Table 3:** Fixed-effect analysis of variance for hemocyte count with parasite source, host source, and parasite dose as factors. R-squared for the model was 0.15.

Source	s.s.	d.f.	m.s.	*F*	*P*
Parasite source	0.257	1	0.257	3.690	0.062
Host source	0.138	1	0.138	1.979	0.167
Dose	0.024	1	0.024	0.347	0.559
Parasite * Host	0.043	1	0.043	0.618	0.436
Parasite * Dose	0.020	1	0.020	0.292	0.592
Host * Dose	<0.001	1	<0.001	0.000	0.989
Parasite * Host * Dose	0.006	1	0.006	0.090	0.766
Error	2.787	40	0.070		
Total	156.99	48			

## Discussion

The results suggest that sympatric, locally adapted parasites (*Microphallus *sp.) induce a similar defense response (as measured by hemocyte concentration) in *P. antipodarum *snails as do allopatric parasites. Although there was a marginally significant main effect for parasite source, there was clearly no host-by-parasite interaction effect on hemocyte count (Fig. 2, Table 2). In contrast, however, the interaction effect was highly significant for infection success; but there were no significant main effects for either host source or parasite source (Fig. 1, Table 1). Therefore, even though the defense was induced to the same degree by both sympatric and allopatric parasites, the defense was less effective against the locally adapted sympatric parasites. One implication of this result is that the snails cannot increase resistance to infection by coevolved trematodes by increasing the allocation to defense.

Hemocyte concentration is only a rough measure of the immune response. However, hemocyte concentration did show a response to parasite exposure in our study (Fig. 2), and defense mechanisms in other snail-trematode systems are mediated through hemocytes [6,7]. For example, hemocytes encapsulate trematodes and excrete reactive oxygen molecules that are cytotoxic [6,8]. In addition, hemocytes up-regulate genes when exposed to trematodes [9,10]. More detailed observation on the reactions of hemocytes in future studies may lead to different results. However, there was no indication that a sympatric or allopatric source of parasites changed the induction of hemocytes in our study (Fig. 3), as was found in Kurtz *et al*. [6] for grasshoppers.

Usually, allocation of resources to defense mechanisms has been found to be costly in terms of survival or reproduction [11-16], and it has been assumed that hosts are trading-off fitness for protection against the negative effects of infection. However, when coevolution changes the effectiveness of the host defense, predictions about trade-offs between parasite defense and other fitness components are more complicated [1]. If local adaptation by parasites decreases the probability that hosts will mount a successful defense, then the optimal defense strategy for the host will depend on both the cost and effectiveness of the defense as well as the virulence of the parasite [1]. In the present study, costs of defense were not measured, but a strong defense response was observed (Fig. 2). Previous work in this system has shown that growth and survival did not decrease with increasing parasite exposure [17], so it is likely that the defense response is not costly or that the cost is small. On the other hand, resources in the lab environment may have been sufficient to compensate for the increased demands of the defense resource. This may have obscured the detection of a difference between host-parasite combinations in the hemocytes response.

If defense is not costly, then mounting a defense would be advantageous whenever the host encounters parasites regardless of the effectiveness of the defense mechanisms. Nonetheless, defense might only be effective against some proportion of the parasite population, as would be expected if there is tight genetic specificity for infection. In such a situation, some level of defense allocation is required to defend against some parasite genotypes, but no level of allocation can defend against other parasite genotypes. This later alternative seems likely given that there is good evidence for local adaptation in this system (Fig. 1) [18-21] and the fact that both allopatric and sympatric combinations induced the same defense response (Fig. 3).

In the present study, hosts seemed to increase defense, perhaps to a maximum, following exposure to a very low dose of parasite eggs, yet they did not increase defense at higher dose (Fig. 2). Therefore, even small exposures may be a sufficient cue that increases defense allocation in environments with a high risk of parasitism. One might expect a dose effect under the assumption that hosts would increase defense levels as more and more parasites were encountered, especially in sympatric host-parasite combinations that are more difficult to defend against. However, there was no effect of parasite dose or any higher order interactions involving dose, host source, or parasite source on hemocyte induction (Fig. 3, Table 3). There was a marginally significant parasite main effect for hemocyte induction (Table 3), meaning that some parasite populations might induce greater hemocyte responses than others. Potentially, coevolution may have increased counter-defenses in some trematode populations; thus, larger allocations to defense are required in the host when confronted with these parasites. Counter-defenses of parasites are known to occur in many systems [22,23]. For example, interference of host hemocyte function has been shown in echinostomes (Trematoda) [24-26], and parasitoids of *Drosophila *bury into the fat body in order to avoid circulating hemocytes [27].

## Conclusion

The interaction is highly specific for infection, but not for defense response as measured by hemocyte concentration. These results are most consistent with a highly specific genetic matching mechanism where successful parasites evade or interfere with defenses of specific host genotypes. There was also no effect of parasite dose on hemocyte concentration after initial exposure, which implies that defense needs do not increase with additional parasite exposures. Although measuring specific aspects of the hemocyte reactions may lead to other results, local adaptation of the parasite to sympatric hosts does not seem to produce variation in the induction of hemocyte concentration across sympatric and allopatric exposures.

## Methods

### Host source

Snail hosts (*Potamopyrgus antipodarum*) were collected from the *Isoetes *habitat at the "Camp" site at Lake Alexandrina, New Zealand, [28] by sweeping a net through the underwater vegetation. Snails from Lake Mapourika were collected by the same methods. Previous studies have shown that parasites from these same two lakes are adapted to infect their sympatric host populations [19]. In addition, in a meta-analysis of multiple studies on this system, we found that 32 of 33 parasite populations (in either space or time) were more successful at infecting local snails than snails collected from allopatric locations [21]. Thus, the pattern of parasite adaptation to local host populations appears to be extremely robust in space and time. Lake Alexandrina is on the eastern side of the Southern Alps at about 750 m above sea level, and Lake Mapourika is on the western side of the Southern Alps at 75 m above sea level [29] on the South Island of New Zealand. Hosts from both sources were collected in January 2002 and transported under permit to the lab at Indiana University in cool and wet paper towels. In the lab the snails were kept in large aquaria and fed Spirulina algae until the experiment began on 2 March 2002. During the experimental trials, each experimental unit consisted of 100 snails kept in a 2 l plastic container. The snails were fed Spirulina algae 1–2 times per week and the water in the containers was changed once a week.

### Parasite system and culture

*Microphallus *sp. (Trematoda) is a widespread and locally common undescribed parasite in New Zealand lakes and streams [28,30]. Multilocus allozyme genotype data show that *Microphallus *is a single outbred species with high levels of gene flow among South Island populations [31]. The parasite exclusively uses *P. antipodarum *as the intermediate host, and the final hosts are waterfowl. Embryonated *Microphallus *eggs are ingested from sediment and hatch in the snail's gut, penetrate the intestine, and migrate to the gonads and digestive gland. Following successful establishment, the parasite then undergoes asexual reproduction, replacing much of the host's reproductive tissue and digestive gland, which results in complete sterilization of the snail. The first visible parasite developmental stages (blastocercariae) are detectable after approximately 75 days post-exposure and metacercariae are common by 90 days post-exposure at 16°C in the lab. The life cycle is completed when snails containing metacercariae are consumed by waterfowl.

Although the natural host for the parasite used in this experiment are birds, the parasite is successfully cultured in mouse [32]. To produce parasite eggs that are infective to snails, three mice were each fed 35 *Microphallus*-infected snails collected from Lake Alexandrina; and similarly, three mice were fed 35 *Microphallus*-infected snails collected from Lake Mapourika. Three additional mice were not fed parasites, and they were used to provide parasite-free feces in one of our control treatments (see below). Starting 24 hours after feeding, the litter in the mice cages was changed, and fecal pellets were collected three times each day for four days. After collection, the control feces and parasite feces were separately placed in fresh pond water in order to store the parasite eggs and dilute soluble waste. The pond water was aerated and changed twice a day.

### Experimental design and procedures

A factorial design was used to study the effects of host source, parasite source, and parasite dose on infection and hemocyte concentration. Thirty 2 l containers each housed 100 snails from Lake Alexandrina, and thirty 2 l containers each housed 100 snails from Lake Mapourika. Within each host source, six containers were given a low dose of Alexandrina parasites, six containers were given a high dose of Alexandrina parasites, six containers were given a low dose of Mapourika parasites, and six containers were given a high dose of Mapourika parasites ("high" and "low" dose were as defined below). The remaining six containers for each host source received no parasites – three containers received mouse feces without parasite eggs and three containers received no parasites or feces. Because there was no difference in hemocyte counts between the no-parasite treatments with feces and the no-parasite treatment without feces (*F *= 0.001, d.f. = 1, 10, *P *= 0.975), these treatments were pooled in all analyses below. This result shows that the contents of the mouse feces were, by themselves, not sufficient to increase the hemocyte counts of snails.

Parasite doses were determined by separately condensing the mixtures of mouse feces and water to 1 l volume. The mixture was then split into 100 ml (low dose) and 900 ml (high dose) amounts and then each was diluted up to 1 l. A serological pipette was then used to administer mouse feces equally among containers for each dose treatment. Thus the high dose was expected to have 9 times as many parasite eggs as the low dose. The feces from control mice were handled in the same way, and they were distributed so that snails in the parasite control with feces and low-dose treatments were exposed to the same amount of mouse feces as the high-dose experimental treatments.

### Hemocyte quantification

Snails were sampled for hemolymph on days 13 and14 post-exposure. From previous experiments (E. Osnas, unpublished data), it was known that hemocyte concentration increases after parasite exposure by this time. At 14 days post-exposure the parasite is visually undetectable in the snail (see above). To collect hemolymph, three individuals were randomly sub-sampled from each experimental container. For each snail, hemolymph was collected by tapping on the operculum until hemolymph was expelled through the hemal pore [13,33,34]. A 10 μl pipette was then used to draw up the hemolymph and transfer it to a hemocytometer. In *P. antipodarum*, this technique results in approximately 1–2 μl of hemolymph. Hemocytes in 0.1 μl of the hemocytometer grid surface were then visually counted using a compound microscope at 400 total magnification.

### Parasite sampling and statistical analysis

After 90 days post-exposure, snails were examined for infection by dissection under a microscope. At 90 days post-exposure, parasites can be easily detected using a dissecting microscope, but they have not reached the fully mature metacercariae stage. Parasite species and developmental stage were recorded [17,35]. Snails with infections of species other than *Microphallus *were excluded from the analysis. Similarly, snails infected by mature *Microphallus *metacercariae were also deleted from the analysis because they were assumed to be already infected when collected in the field [17,36].

For each container, we calculated the percent infection (prevalence) of the snails sampled after 90 days post-exposure, and we calculated the average hemocyte count for the three individuals sampled after 13 days post-exposure. These means were then used as independent data points for statistical analysis. We used a three-factor fixed effect analysis of variance with host source, parasite source, and parasite dose as independent factors to test for local adaptation by the parasite. For this analysis, the control treatments (no parasites) were excluded. Local adaptation of the parasite to the host is indicated by a significant crossing-type interaction between host and parasite source.

We used one-way analysis of variance in order to test for a change in hemocyte concentration with parasite exposure. For this analysis, dose was the independent variable and log-base 10 of hemocyte count was the dependant variable. We used three levels of dose: no exposure, low dose, and high dose, as describe above. We then used independent contrasts to test for an overall increase in hemocyte count with exposure (control verses low and high) and for a difference between low and high exposure levels.

To test for an effect of parasite source, host source, and dose on hemocyte count, we used a full factorial three-factor fixed effect model analysis of variance, just as above for infection. For this analysis, the control treatments (no parasites) were also removed from the data set. This produced a balanced design. All analyses were done in SPSS 12.0 (2003).

## Authors' contributions

EEO and CML developed the concept and experimental design for this study. EEO preformed the experiment, collected the data, and wrote a first draft of the manuscript. CML helped perform the experiment and substantially revised the manuscript. Both authors read and approved of the final manuscript.
